# Association of microRNAs with Argonaute proteins in the malaria mosquito *Anopheles gambiae* after blood ingestion

**DOI:** 10.1038/s41598-017-07013-1

**Published:** 2017-07-26

**Authors:** Xiaonan Fu, George Dimopoulos, Jinsong Zhu

**Affiliations:** 1 The Interdisciplinary PhD Program in Genetics, Bioinformatics, and Computational Biology, Virginia Tech, Blacksburg, Virginia USA; 2 W. Harry Feinstone Department of Molecular Microbiology and Immunology, Bloomberg School of Public Health, Johns Hopkins University, Baltimore, Maryland USA; 3 Department of Biochemistry, Virginia Tech, 340 West Campus Drive, Blacksburg, Virginia 24061 USA

## Abstract

Drastic changes in gene expression occur after adult female mosquitoes take a blood meal and use the nutrients for egg maturation. A growing body of evidence indicates that microRNAs (miRNAs) contribute to this tightly controlled tissue- and stage-specific gene expression. To investigate the role of miRNAs, we monitored miRNA expression in the mosquito *Anopheles gambiae* during the 72-h period immediately after blood feeding. We also measured the association of miRNAs with Argonaute 1 (Ago1) and Argonaute 2 (Ago2) to assess the functional status of individual miRNA species. Overall, 173 mature miRNAs were precipitated with Ago1 and Ago2, including 12 new miRNAs, the orthologs of which are found thus far only in other *Anopheles* species. Ago1 is the predominant carrier of miRNAs in *Anopheles gambiae*. The abundance and Ago loading of most of the mature miRNAs were relatively stable after blood ingestion. However, miRNAs of the miR-309/286/2944 cluster were considerably upregulated after blood feeding. Injection of the specific antagomir for miR-309 resulted in smaller developing oocytes and ultimately fewer eggs. In addition, the Ago association of some miRNAs was not proportional to their cellular abundance, suggesting that integration of miRNAs into the Ago complexes is regulated by additional mechanisms.

## Introduction

The *Anopheles gambiae* mosquito is the major vector of human malaria in Africa. Malaria is caused by protozoan parasites belonging to the genus *Plasmodium* and is spread between hosts by the bites of parasite-infected female mosquitoes. Female adults of *An*. *gambiae* must obtain blood from vertebrates to acquire nutrients for egg production. The female mosquitoes digest a protein-rich blood meal, greater than their original body weight, within 48 h after ingestion and use the nutrients to synthesize egg yolk proteins. Substantial physiological changes occur after blood ingestion in mosquitoes, and over 50% of all genes show significant variation in transcript accumulation^1^. Recent studies suggest that blood digestion and egg production in *An*. *gambiae* and other mosquito species are also regulated by mosquito microRNAs (miRNAs)^2^. Expression of some miRNAs is considerably altered after blood ingestion in *An*. *gambiae* ^3^, *An*. *stephensi* ^4^, *Aedes aegypti* ^5^, and *Aedes albopictus* ^6^. Functional studies in *Ae*. *aegypti* have shown that perturbation of individual miRNAs in female mosquitoes leads to defects in blood digestion and egg production^7–10^.

miRNAs are small endogenous non-coding RNAs that play important roles in post-transcriptional gene regulation in plants and animals^11^. Most miRNAs are transcribed by RNA polymerase II as primary-miRNA (pri-miRNA) transcripts. The canonical miRNA pathway converts the pri-miRNA hairpins into ∼22-nucleotide (nt) mature duplex miRNAs via consecutive cleavages by two RNase III enzymes, Drosha and Dicer, with the help of some double-stranded RNA-binding proteins^12^. The two strands of the mature miRNA duplex are denoted by the suffix -5p or -3p, depending on whether they originate from the 5′ or 3′ end of the pri-miRNA. Only one strand of the miRNA duplex is usually loaded onto an Argonaute (Ago) protein as part of the miRNA-induced silencing complex (miRISC). The strand in miRISC is known as the guide strand; the other strand (the passenger strand) is excluded and subsequently degraded^12^. The guide strand directs the miRISC to messenger RNA (mRNA) targets via imperfect base pairing, leading to reduced gene expression through mRNA destabilization and/or translational repression^13^. However, recent studies have demonstrated that the 5p and 3p strands of some miRNAs are expressed in comparable concentrations, and both strands seem to have biological functions^14–17^. To complicate matters further, the association of individual miRNA species with Ago proteins is not always proportional to their cellular levels, implying that integration of miRNAs into RISC is regulated by additional mechanisms^18–21^.

The five Argonaute proteins in the fruit fly *Drosophila melanogaster* fall into two subfamilies. The AGO subfamily consists of Ago1 and Ago2, and the Piwi subfamily consists of Piwi, Aubergine (Aub), and Ago3^13^. *Drosophila* miRNAs are preferentially loaded into Ago1, whereas small interfering RNAs (siRNAs) are mainly incorporated into Ago2^22–24^. Piwi proteins and the Piwi-interacting RNAs (piRNAs) are involved in germline development, epigenetic regulation of gene transcription, transposon silencing, mRNA turnover, and translational control^25^. Comparative phylogenetic analysis has revealed two AGO subfamily members in the mosquito *An*. *gambiae*: AgAgo1 and AgAgo2^26^. While experimental evidence has shown that AgAgo1 and AgAgo2 play critical roles in the miRNA pathway and the siRNA pathway, respectively, small RNA partition between Ago1- and Ago2-RISC has not been carefully examined^3, 27^.

In the present study, we performed a systematic analysis of RNA contents that were associated with Ago1 and Ago2 at multiple time points in blood-fed *An*. *gambiae*. Our results confirmed that miRNAs were mostly enriched in the Ago1-RISC in *An*. *gambiae*, while siRNAs primarily occupied Ago2. Importantly, we found that the vast majority of mosquito miRNAs maintained their associations with Ago1 and Ago2 at relatively constant levels during the 72 h immediately after blood ingestion. miR-309 was among a small set of miRNAs that displayed an enhanced integration into Ago1 after a blood meal. Depletion of miR-309 significantly reduced mosquito egg production, implying that the extent of Ago1 loading might be used to evaluate the inhibitory potential of specific miRNAs in a biological process.

## Results

### Deep sequencing of small RNAs associated with Ago1 and Ago2

To use RNA immunoprecipitation for the isolation of Ago-bound small RNAs in *An*. *gambiae*, we produced polyclonal antibodies separately for AgAgo1 and AgAgo2. The specificity of the antigen-purified antibodies was validated by immunoprecipitation of the AgAgo1-V5 and AgAgo2-V5 fusion proteins that were synthesized *in vitro*. The Ago antibodies only recognized their corresponding fusion proteins and did not exhibit any cross-reactivity to the other Ago proteins (Supplementary Fig. S1A). Similar results were observed when immunoprecipitation experiments were performed to detect the Ago proteins extracted from adult mosquitoes (Supplementary Fig. S1B).

**Figure 1:**
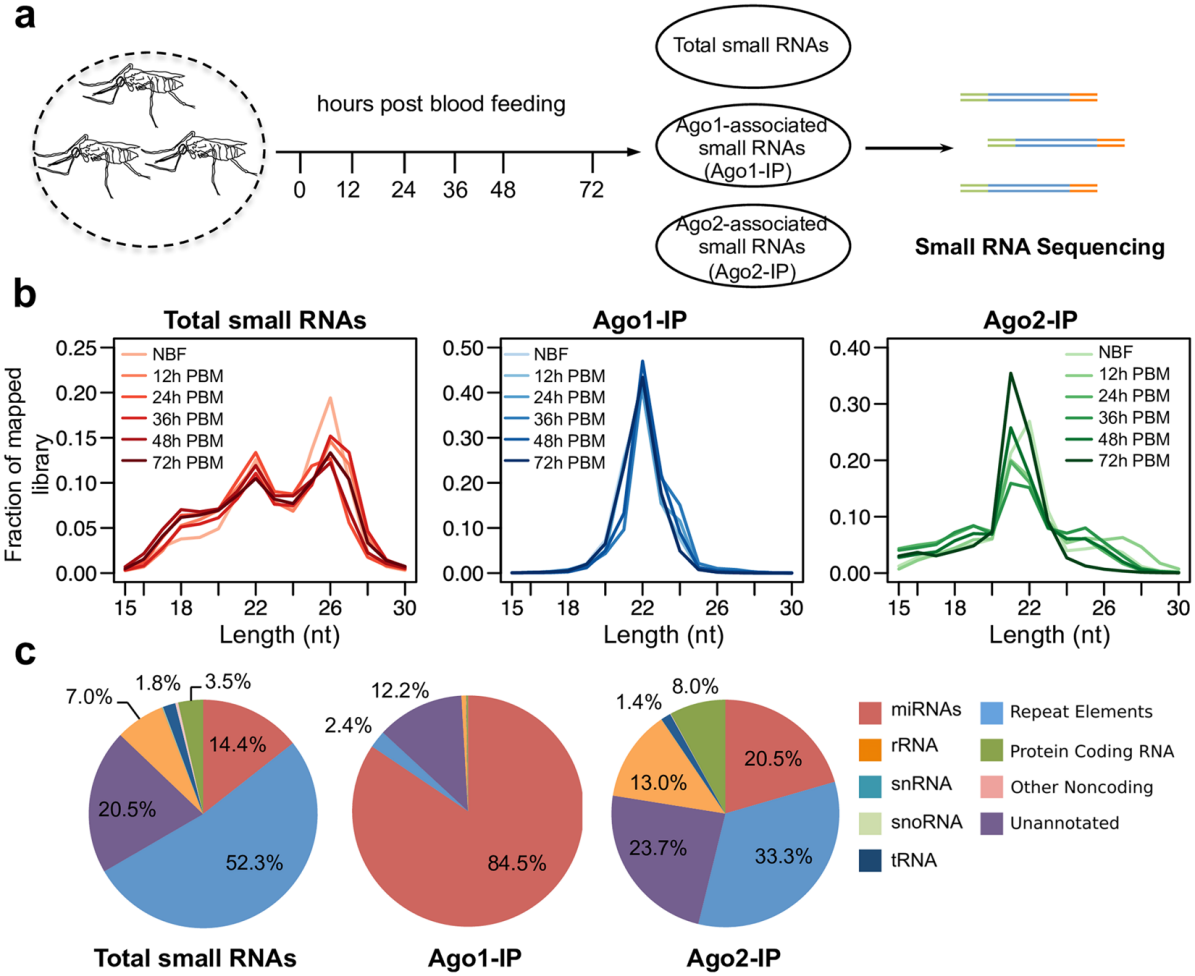
Sequencing of *An*. *gambiae* small RNAs. (**a**) Schematic of small RNA library preparation. Adult abdomens were dissected from female *An*. *gambiae* at the indicated time points. Three types of RNA pools (Total, Ago1-IP and Ago2-IP) were prepared independently. Small RNAs of 15–30 nt were size-selected from 10% TBE-urea gels and purified for library construction. (**b**) Length distribution of total sequencing reads. NBF, non-blood-fed; PBM, post-blood meal. (**c**) Annotation of sequencing reads. Reads from the same type of RNA libraries were put together for comparison. Minor fractions (less than 1%) are not labeled.

Dynamic gene expression takes place in female *An*. *gambiae* upon blood ingestion, and female adults are normally ready to lay eggs at about 72 h after blood feeding. To systematically investigate the Ago-miRNA interaction in *An*. *gambiae*, we collected abdomens from adult female mosquitoes at 0, 12, 24, 36, 48, and 72 h after a blood meal (PBM). For each time point, we isolated three discrete RNA populations: total cellular small RNAs, small RNAs associated with AgAgo1 (Ago1-IP), and small RNAs associated with AgAgo2 (Ago2-IP) (Fig. 1A). A total of 18 small RNA libraries were constructed from samples collected at the six time points for Illumina high-throughput sequencing.

We obtained 42.88 million raw reads in total from the sequencing data. After adapter removal and quality filtering, 38.15 million sequence reads were retained for genome mapping. About 84–96% of the total reads from individual libraries were mapped to the *An*. *gambiae* genome (AgamP4, VectorBase) (Supplementary Table S1). The sequencing data have been submitted to the NCBI SRA database (accession number: SRP101587). To assess the quality of the normalized datasets, ten miRNAs were randomly chosen, and their relative abundance was validated by quantitative reverse-transcription PCR. The high correlation coefficients (R = 0.92 for total small RNA pools; R = 0.94 for Ago1-IP RNA pools) indicated a reliable miRNA measurement by deep sequencing (Supplementary Fig. 2A). In addition, the quality and integrity of the miRNA dataset was assessed using principal component analysis (PCA). Excellent segregation was apparent for the miRNAs in the total small RNA pools, the Ago1-IP pools, and the Ago2-IP pools, reflecting the high integrity of the samples (Supplementary Fig. 2B). While the total cellular miRNA pools or the Ago2-IP miRNA pools displayed considerable closeness at the six time points, the Ago1-IP miRNA pools showed more distance between each other, suggesting more temporal variation in the loading of the miRNAs onto Ago1 after blood feeding.

The 18 small RNA libraries had a size distribution between 15 and 30 nt (Fig. 1B). Total small RNAs from all six time points displayed three peaks, presumably representing distinct classes of small RNAs. The small RNAs with a peak at 18 nt were likely the transcription initiation RNAs (tiRNAs) that are found in humans, chickens, and flies^28^. tiRNAs are derived from sequences immediately downstream of RNA polymerase II (RNAPII) transcription start sites and are connected to RNAPII binding and epigenetic regulation^28^. Mapping of the 18-nt RNA reads from our total small RNA libraries indeed showed substantial enrichment near the transcription start sites (Supplementary Fig. 3). The peaks at 22 nt matched to the typical length of mature miRNAs. A population of small RNAs, ranging from 24 to 30 nt in length with a peak at 26 nt, were previously reported to represent piRNAs in *D*. *melanogaster* ^29^ and *Ae*. *aegypti* ^30^. In contrast, the small RNAs associated with Ago1 showed a single prevalent peak at 22 nt, and the Ago2-associated RNAs gave a distribution peak at 21–22 nt (Fig. 1B). The distinct size distribution across different types of libraries implies that our separation of RNAs was effective and consistent.

### Identification and validation of novel *An*. *gambiae* miRNAs

All the mapped reads were then successively annotated with miRBase, repeat annotations, Rfam, and RefSeq mRNAs (AgamP4.0, VectorBase) to identify miRNAs, repeats, noncoding RNAs, and *An*. *gambiae* mRNAs. Comprehensive analysis of RNAs extracted from the AGO complexes revealed 173 unique mature miRNAs derived from 108 miRNA loci of *An*. *gambiae* (Supplementary Table S2). The mature miRNAs included 128 annotated *An*. *gambiae* miRNAs in miRBase (Version 21), 32 additional mature miRNAs reported by Biryukova *et al*.^3^, and 13 novel miRNAs discovered in the current study (Table 1). We followed a set of general rules to filter putative miRNAs from all the unclassified reads: 1) the predicted pre-miRNA formed a stable hairpin structure; 2) at least 20 sequence reads from more than one of the Ago-IP libraries were perfectly aligned to the predicted pre-miRNA hairpin; 3) a vast majority of the reads were mapped to one or two arms of the pri-miRNA.

**Table 1:** Novel miRNAs in *An*. *gambiae*.

Seq_ID	Chromosome	Start	End	Strand	Arm	Mature Sequence	Energy (kcal/mol)
miR-N1	2 L	37758152	37758241	−	3p	CUCAUCAAUUGGUUGUGGCUAUG	−26.30
miR-N2	3 L	17216211	17216296	+	5p	AUUAGAAUGUGGAAUCUGUUUU	−22.30
miR-N3	Unknown	30034502	30034587	−	3p	UGACUUGUCUGCCUGAGCAAGGGG	−32.70
miR-N4	2 R	36376058	36376154	−	3p	AAGACGCUGGUUCUCUUCACACA	−27.50
miR-N5	×	1722538	1722623	−	3p	UUGGGCGCUAUCUACAAUGUAG	−26.66
miR-N6	Unknown	30034504	30034585	−	3p	UAUGACUUGUCUGCCUGAGCAAGG	−32.70
miR-N7	Unknown	9226296	9226379	−	3p	GUGGAACCGCGUAGGCUGCCU	−22.90
miR-N8	×	23502516	23502590	+	3p	UGACGGGUUUGGUCUCUCCA	−57.50
miR-N9	2 L	48562288	48562365	+	5p	UAGUCGUUUUCUGCUUUGCGGUU	−20.30
miR-N10	3 R	44916363	44916443	−	3p	UGUUCGAUCGUUACUGUCAUAU	−24.00
miR-N11	2 R	18852629	18852719	−	5p	CGUGGUACUCUUGUGGUAAGG	−39.20
miR-N12	2 R	7197866	7197955	−	3p	UGCAUUCAGUGGGGCGGUCGUG	−42.89
miR-N13	3 L	7171866	7171950	−	5p	AGCUGUUCUGACUUGAUGUACU	−28.31

We performed conservation analysis to search for the orthologs of the novel miRNAs in the genomes of *D*. *melanogaster*, *Ae*. *aegypti*, *Ae*. *albopictus*, *Culex quinquefasciatus*, and 19 additional *Anopheles* species. The pre-miRNA sequences were mapped to the genomes by reciprocal BLAST searches. None of these novel miRNAs could be found in *D*. *melanogaster*. The ortholog of aga-miR-N2 existed in *Ae*. *aegypti*, *Ae*. *albopictus*, *C*. *quinquefasciatus*, and a few *Anopheles* species, implying that it is a conserved miRNA in mosquitoes. The other novel miRNAs had their putative orthologs in only some of the 20 *Anopheles* species with published genome sequences, suggesting that they are *Anopleles*-specific miRNAs (Table 2). aga-miR-N5 was of particular interest, since it has been found thus far only in *An*. *gambiae*. The mature aga-miR-N5 and its star strand were both detected in the RNA purified by immunoprecipitation of the AGO complexes (Supplementary Fig. S4). Expression of this *An*. *gambiae*-specific miRNA was further validated by quantitative RT-PCR (qRT-PCR). In adult female mosquitoes, the levels of aga-miR-N5 decreased after blood ingestion, reached a nadir at 36 h PBM, and gradually rose after that (Supplementary Fig. S4).

**Table 2:** Conservation of novel miRNAs in 20 *Anopheles* species.

miRNA	An. albim-anus	An. arabi-ensis	An. atrop-arvus	An. christyi	An. coluzzii	An. culici-facies	An. darlingi	An. dirus	An. epiro-ticus	Anop-heles-farauti	An. fune-stus	An. gam-biae	An. macu-latus	An. melas	An. merus	An. mini-mus	An. nili	An. quadria-nnulatus	An. sine-nsis	An. stephensi
aga-mir-N1	×	×	×	×	×	×	×	×	×	×	×	×	−	×	×	×	−	×	×	×
aga-mir-N2	×	×	×	−	×	×	×	×	×	×	×	×	×	×	×	×	×	×	×	×
aga-mir-N3	−	×	−	−	×	−	−	−	−	−	−	×	−	−	×	−	−	×	−	−
aga-mir-N4	×	×	×	×	−	×	×	×	×	×	×	×	−	×	×	×	×	×	×	×
aga-mir-N5	−	−	−	−	−	−	−	−	−	−	−	×	−	−	−	−	−	−	−	−
aga-mir-N6	−	×	−	−	×	−	−	−	−	−	−	×	−	−	×	−	−	×	−	−
aga-mir-N7	−	×	−	−	×	-	−	−	−	−	−	×	−	×	×	−	−	×	−	−
aga-mir-N8	−	×	−	−	−	−	−	−	−	−	−	×	−	−	−	−	−	−	−	−
aga-mir-N9	−	×	−	−	×	−	−	−	−	−	−	×	−	×	×	−	−	×	−	−
aga-mir-N10	−	×	−	×	×	−	−	×	−	−	×	×	−	×	×	×	−	×	−	×
aga-mir-N11	×	×	×	×	×	×	×	×	×	×	×	×	−	×	×	×	−	×	×	×
aga-mir-N12	×	×	×	×	×	×	×	×	×	×	×	×	×	×	×	×	−	×	×	×
aga-mir-N13	−	×	−	−	×	−	−	−	−	−	−	×	−	×	×	−	−	×	−	−

Note: “×” indicates that the miRNA orthologue is found by reciprocal blast; “−” means no hits.

### Sorting of miRNAs into Ago1 and Ago2

Analysis of the RNA dataset revealed miRNAs and small RNAs derived from non-miRNA sources, including transfer RNA (tRNA), small nuclear RNA (snRNA), small nucleolar RNA (snoRNA) and mRNA, in all the small RNA libraries. miRNAs constituted an average of 14.4% of the mapped reads from the libraries prepared with total small RNA (Fig. 1C). In the Ago1-IP libraries, miRNAs were the dominant class of RNA, accounting for 84.5% of the mapped reads on average (Fig. 1C). The increased miRNA abundance in the Ago1-IP RNA libraries indicated that miRNAs were enriched by immunoprecipitation of Ago1. The miRNA fraction made up an average of 20.5% of the mapped RNA-seq reads in the Ago2-IP libraries (Fig. 1C). In addition, 8.0% of the Ago2-IP RNA reads mapped to the protein-coding genes, 23.7% mapped to unannotated regions, and 33.3% mapped to repetitive and transposable elements, constituting a large set of endogenous siRNAs that have previously been reported in *An*. *gambiae* ^31^. Therefore, Ago1 in *An*. *gambiae* is the main carrier of miRNAs, while Ago2 is preferentially loaded with siRNAs, although some miRNAs are also sorted into Ago2.

**Figure 2:**
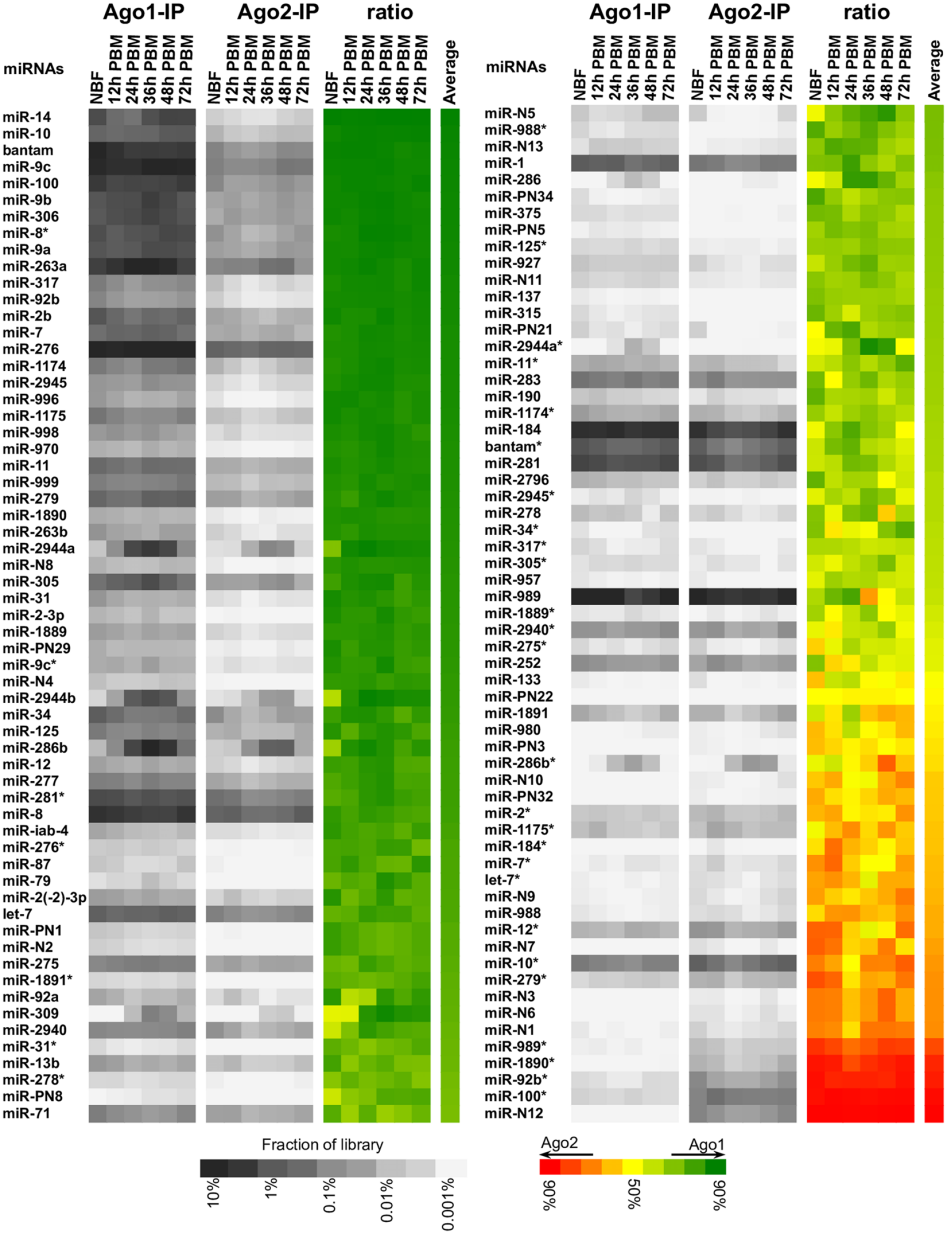
Integration of miRNA and miRNA* strands into Ago1 and Ago2. Heat maps illustrate the relative abundance of miR and miR* in the individual Ago1-IP and Ago2-IP libraries (grayscale). The ratio of normalized representation in the libraries indicates the relative loading of each miR or miR* strand with Ago1 (green) or Ago2 (red).

After the miRNA duplex is generated by Dicer 1, only one strand of the duplex is retained and integrated into miRISCs. The other strand is usually degraded by an unknown mechanism^12^. Asymmetric selection of the duplex often produces a mature miRNA (miR) of higher abundance at steady-state and a less frequently detected strand that is historically referred to as miRNA star (miR*)^32^. In *Drosophila*, over 90% of the Ago1-associated reads are mapped to the miR strand, while about 60% of the Ago2-associated miRNAs are annotated as miR*^33^. Our sequencing data from Ago1 and Ago2 immunoprecipitation enabled us to explore the strand partitioning of miR/miR* duplexes in *An*. *gambiae*. Since the miR and miR* of *An*. *gambiae* were not fully curated in miRBase, we annotated the miR and miR* strands based on their relative abundance in the total RNA pools. miR* strands accounted for 6.2%, 5.0%, and 10.3% of miRNAs in the total small RNAs, Ago1-IP RNAs, and Ago2-IP RNAs, respectively (Fig. 2). The *An*. *gambiae* miR* strands were significantly over-represented (p < 0.00001) in the Ago2-associated small RNA sequences, but to a lesser extent than the strong preferential loading of Ago2 with miR* in *D*. *melanogaster*.

The data collected at multiple time points after blood digestion were also used to examine the strand selection under different conditions. To ascertain the Ago loading pattern of individual miRNA, the relative abundance of each miRNA in the AGO complexes was measured in terms of its normalized transcripts per million reads (TPM). The miR and miR* strands of many miRNAs were detected in both the Ago1 complexes and the Ago2 complexes (Fig. 2). The miR strands of a majority of the miRNAs were predominantly associated with Ago1. However, a small group of miRNAs, including several novel miRNAs discovered in this study (miR-N1, miR-N3, miR-N6, miR-N7, miR-N9, miR-N10, and miR-N12), primarily loaded their miR strands onto Ago2 (Fig. 2). The miR and miR* strands of a few other miRNAs (e.g., bantam and miR-8) were present in comparable levels in the Ago1 complex. Our Fisher’s exact test indicated that 19 of the 33 miR*s detected in this study were significantly enriched (p ≤ 0.01) in the Ago2-IP RNA. Strand selection of individual miRNAs was generally unchanged within the 72 h immediately after blood feeding (Fig. 2).

### Structural difference between miRNAs associated with Ago1 and Ago2

**Figure 3:**
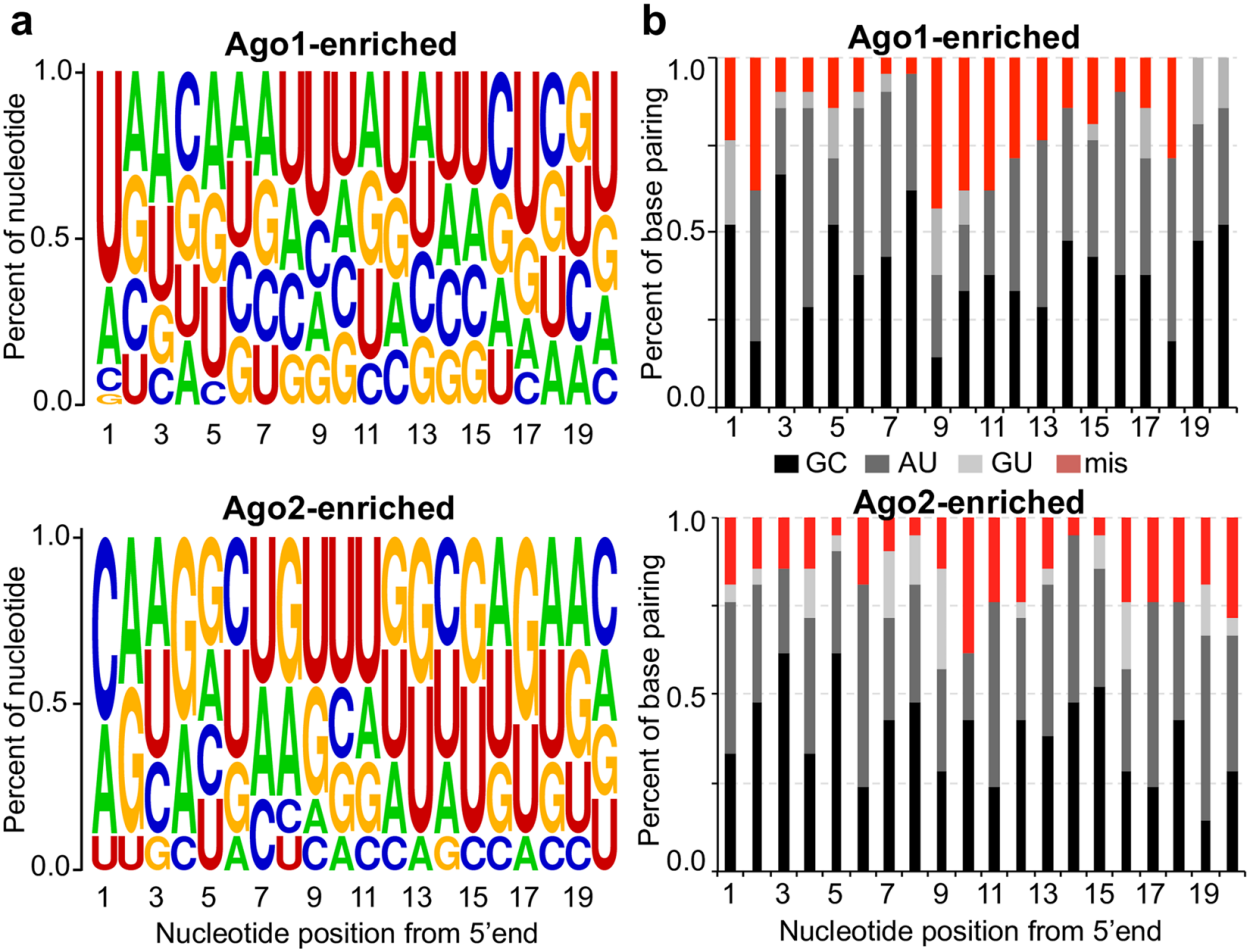
Structural determinants of the Ago1 and Ago2-enriched miRNAs. (**a**) Nucleotide composition of miRNAs that displayed strong preferential (>70%) association with Ago1 or Ago2. The y axis is the frequency of each nucleotide at each position. (**b**) Median base pairing at each position of all miRNAs that showed a highly biased (>70%) association with Ago1 or Ago2. Bulges on each strand were counted as mismatches (mis).

Studies of the miRNA-Ago interaction in *Drosophila* have shown that the 5′ terminal nucleotide composition and the central mismatch in pre-miRNA duplex contribute to the sorting decision^32, 33^. To test whether the same rules apply to miRNAs in *An*. *gambiae*, we chose miRNAs that exhibited strong biased (>70%) association with either Ago1 or Ago2 to perform a comparative study. In the Ago1-bound miRNAs, there was a prominent enrichment of a 5′-uridine (5′-U) (U at 64.0%, p = 5.919e-19) (Fig. 3A). Conversely, the first nucleotide of the Ago2-loaded miRNAs was more likely a cytidine (C at 55.6%, p = 0.00847), similar to the terminal nucleotide biases observed in the miRNAs associated with *Drosophila* Ago1 and Ago2^33^.

To evaluate the effect of overall base-pairing patterns and the distributions of mismatches within miR:miR*, we calculated the base-pairing probability of mature miRNA at each nucleotide position (Fig. 3B). miRNAs that were preferentially sorted into Ago1 were more likely to have an unpaired 5′ end (position 2) and a central mismatch at position 9. In contrast, the Ago2-enriched miRNAs generally had more mispairing at the 3′ end (positions 19 and 20) than the Ago1-bound miRNAs.

### miRNA sequence heterogeneity and Ago-miRNA association

miRNA sequence variations (also called iso-miRs) exist in many species as a result of inaccurate Drosha and/or Dicer processing, as well as post-transcriptional trimming/tailing. Recent studies of miRNA turnover suggest that non-templated nucleotide additions at the 3′ end of miRNAs (3′ tailing) increase or decrease miRNA stability^34, 35^. We carefully examined miRNA sequence variation in the 18 *An*. *gambiae* small RNA libraries. The 5′ end of all the miRNAs showed strong homogeneity in all the samples (Supplementary Fig. S5), presumably resulting from a highly accurate cleavage by Drosha/Dicer and selection pressure to maintain a conserved seed region. Over 30% of the miRNA reads in each library displayed some sort of trimming or addition to the predominant mature miRNAs at the 3′ end (Supplementary Fig. S5). The variations included both template-directed and non-template modifications. Ago2 appeared to be loaded preferentially with miRNAs that contained a one-nucleotide addition at the 3′ end. In the total small RNA pools and the Ago1-IP RNAs, fewer than 10% of the miRNAs, on average, harbored this nucleotide addition at the 3′ end. In contrast, about 35% of the miRNAs in Ago2 complexes displayed this addition in all 6 Ago2-IP libraries, indicating a consistent bias (Supplementary Fig. S5). Although many miRNAs displayed similar sequence variations in both the Ago1 complex and Ago2 complex, certain variations of some miRNAs occurred predominantly in only one of the Ago complexes (Supplementary Fig. S6). For example, the template-directed variants of miR-281-5p were loaded mainly onto Ago1. In contrast, two variants of miR-989-3p with length divergence were detected in adult mosquitoes at all the time points. The predominant 22-nt form was exclusively associated with Ago1, while ~90% of the miR-989-3p loaded on Ago2 was of the minor 23-nt form (with an extra cytidine at its 3′ end) (Supplementary Fig. S7).

### Abundance and Ago loading of miRNAs after blood ingestion

To study miRNA abundance and integration into the Ago complexes, we selected 122 miRNAs that were expressed at a level of >20 transcripts per million reads (TPM) in at least one of the 18 RNA libraries. Relative levels of miRNAs in the total small RNA pools from the blood-fed mosquitoes were compared with the basal levels in the non-blood-fed control (Supplementary Table S3). Three major clusters were identified by unsupervised clustering analysis (Supplementary Fig. S8). Cluster I contained a single miRNA (miR-92a), the expression of which peaked at 12 h after blood ingestion. miRNAs in Cluster II displayed elevated expression at 36 h and 48 h PBM; their expression fell to near the pre-meal levels at 72 h PBM. The miRNA abundance of Cluster III, which encompassed 73 miRNAs, decreased after blood feeding (Supplementary Fig. S8). In our study, 44 miRNAs from the total RNA pools exhibited >4-fold changes at one or more time points after blood feeding (Supplementary Table S4). The six miRNAs showing enhanced expression were all from the miR-309/miR-2944/miR-286 cluster on chromosome 3 R and its fragmented duplication on chromosome 2 L, which contains only mir-2944a and mir-286b^3^. The enhanced expression was validated in three independent biological replicates by quantitative RT-PCR (Supplementary Fig. S9).

**Figure 4:**
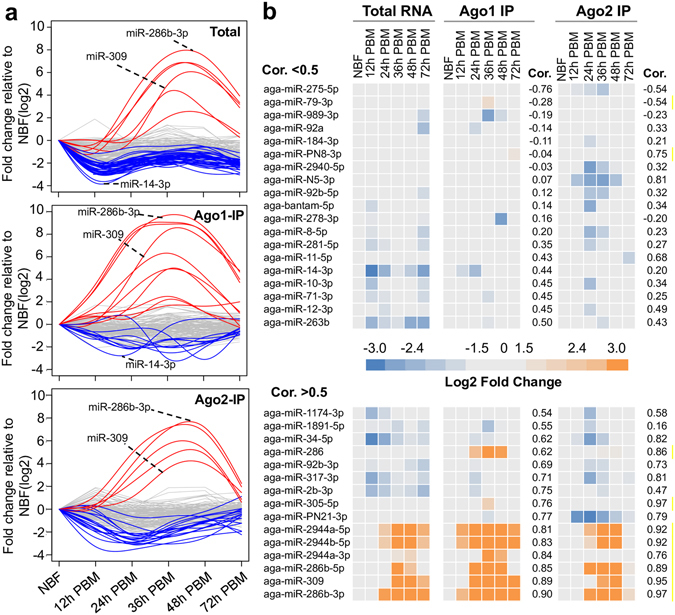
Expression and Ago integration of miRNAs after blood feeding in *An*. *gambiae*. (**a**) Changes in miRNA levels after blood feeding in terms of total small RNA, Ago1-IP RNA and Ago2-IP RNA. Each line represents one miRNA at six time points. Lines in red: upregulated miRNAs; lines in blue: downregulated miRNAs; lines in gray: miRNAs with no significant change. (**b**) Heatmap shows the log2-fold changes in miRNAs relative to the non-blood-fed controls. A Pearson correlation analysis were performed to assess the relationship between miRNA abundance in the total small RNA and in the Ago-IP RNA. Cor., Pearson correlation coefficient.

In parallel, we examined the association of miRNAs with Ago1 and Ago2 over the time course. Hierarchical clustering revealed distinct patterns for the miRNA integration into Ago1 or Ago2 during the 72 h after blood feeding (Supplementary Fig. S8). Ten miRNAs manifested increased association (>4-fold) with Ago1 at one or more time points, whereas 11 miRNAs showed a reduced association with Ago1 (Supplementary Table S4, Fig. 4A). For the interaction with Ago2, the loading of five miRNAs was enhanced (>4-fold), and the integration of 19 miRNAs into Ago2 was weakened at at least one time point (Fig. 4A).

The association of miRNAs with Ago proteins frequently occurred in sync with their cellular abundance. After blood feeding, the miR-309/miR-2944/miR-286 cluster exhibited a considerable increase in their association with Ago1 and Ago2 as well as an increase in total cellular levels (Fig. 4B, Supplementary Fig. S9). miR-2b and miR-317, on the other hand, showed a marked decrease in their concentrations and in their binding to Ago1 and Ago2 after blood ingestion. In some cases, the Ago loading of miRNAs clearly did not match the miRNA abundance. Compared with the non-blood-fed mosquitoes, miR-92a, miR-989-3p and miR-278-3p were less associated (>4 fold, *p* < 0.01) with Ago1 at 24 h, 36 h, and 48 h PBM, respectively, when their total cellular levels remained largely unchanged (Fig. 4B, Supplementary Fig. S9). In contrast, the cellular levels of miR-10-3p and miR-1174-3p decreased by >4-fold at 12 h PBM, but their association with Ago1 did not change accordingly. These discrepancies suggest that the amounts of miRNAs associated with Ago1 may be a more accurate measurement of functional miRNAs. It is important to note, however, that the current RNA-seq study did not include biological replicates because of the large number of discrete RNA populations. Although the abundance and Ago loading of selected miRNAs were mostly validated by qRT-PCR (Supplementary Fig. S9), more rigorous statistical analysis with proper replicates would yield more reliable data on differential miRNA expression and Ago loading.

### Functional analysis of miRNAs that altered their association with Ago1 after blood feeding

**Figure 5:**
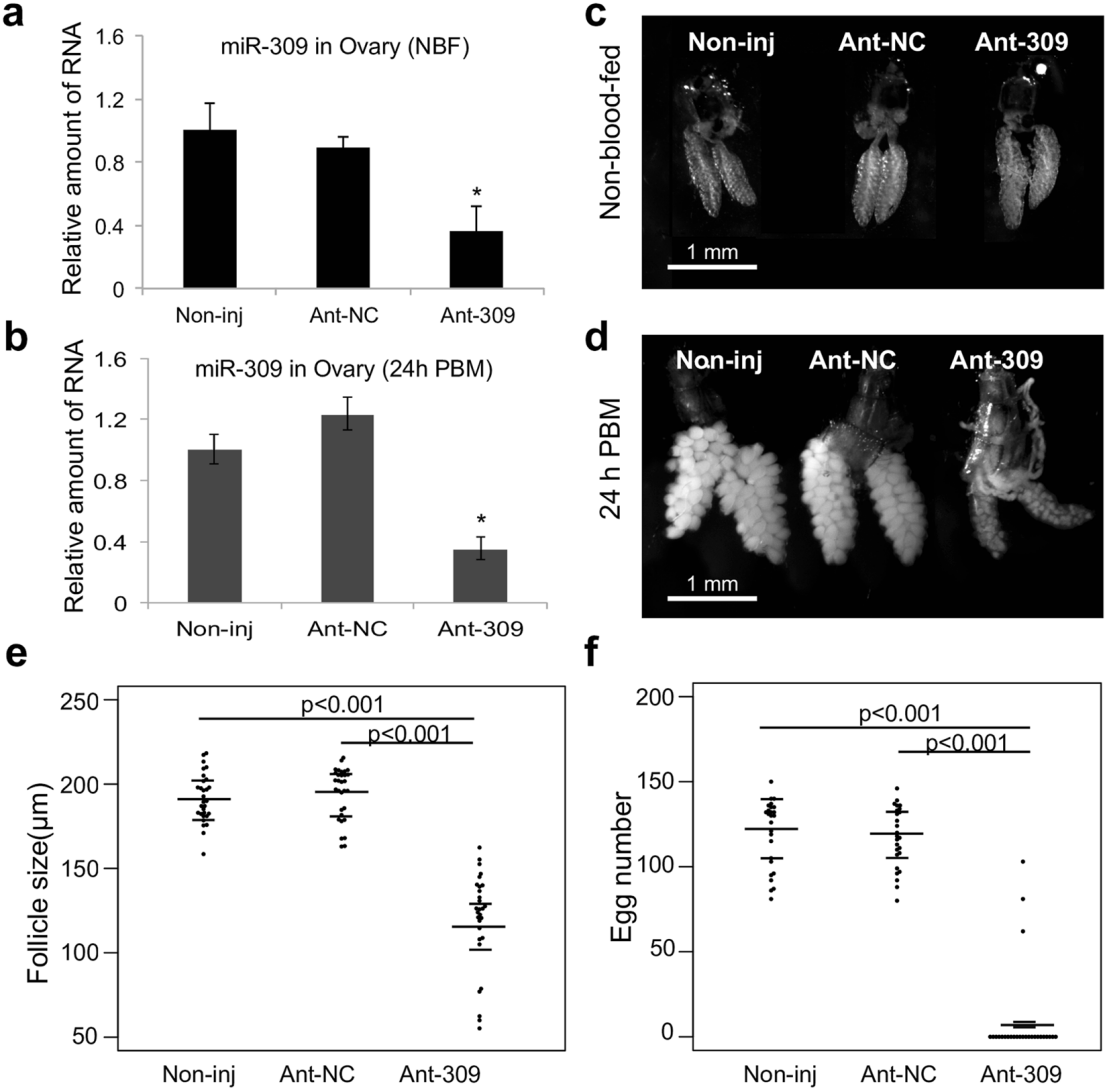
miR-309 is essential for normal egg development. Specific (Ant-309) and control (Ant-NC) antagomirs were injected into adult female mosquitoes at 12 h after adult emergence. The levels of miR-309 in mosquito ovaries were then measured by quantitative RT-PCR 5 days after emergence (before taking a blood meal) (**a**) and at 24 h PBM. (**b**). Oocyte development in the antagomir-treated mosquitoes was compared before blood ingestion (**c**) and at 24 h PBM (**d**). (**e**) Follicle sizes of the noninjected (Non-inj), the Ant-NC-treated, and Ant-309-treated mosquitoes at 24 h PBM (n = 30). (**f**) Egg numbers per female mosquito after a blood meal for the noninjected, the Ant-NC-treated, and Ant-309-treated mosquitoes (n = 30).

Among the miRNAs showing enhanced integration into Ago1 after blood feeding were the miR-309/miR-2944/miR-286 cluster. To elucidate the functional role of these miRNAs in mosquitoes, we predicted their mRNA targets by using three target prediction tools: miRanda^36^, RNAhybrid^37^ and TargetScan^38^. Target sites that were predicted by at least two algorithms were retained for further analysis. Enrichment analysis of Gene Ontology (GO) terms suggested that the putative targets of the miR-309/miR-2944/miR-286 cluster were primarily involved in development and various metabolic processes (Supplementary Fig. 10). To explore the functions of these miRNAs, we injected specific antagomirs into adult female *An*. *gambiae* mosquitoes at 12 h after eclosion. The levels of miR-309 were brought down at 5 days after eclosion and at 24 h PBM by the antagomir-309, but not by a scrambled control antagomir (Fig. 5). In mosquitoes depleted of miR-309, the follicles failed to increase in size after a blood meal, indicating that the development of oocytes was stalled. Concomitantly, very few eggs were laid by the antagomir-309-treated mosquitoes, while the control antagomir showed no adverse effect on egg production. This result indicated that miR-309 plays an important role in regulating mosquito egg maturation. Inhibition of miR-2944 and miR-286 by their cognate antagomirs was verified by qRT-PCR, but clear phenotypic change has not yet been observed.

## Discussion

Argonaute proteins are key effectors in post-transcriptional gene silencing pathways. The number and function of Ago proteins can vary greatly across animal species. Flies have a strict small-RNA sorting system; miRNA duplexes and siRNA duplexes are sorted into Ago1 and Ago2, respectively^22–24^. In humans, all four Ago proteins can integrate both siRNA and miRNA duplexes; miRNAs are randomly sorted to individual Ago proteins without clear preference^39^. The functions of Ago1 and Ago2 in mosquitoes have been extrapolated from studies of flies and are also supported by RNAi experiments in mosquitoes^3, 27^. In the present study, we isolated small RNAs associated with *An*. *gambiae* Ago1 and Ago2. This approach allows us to determine authentic mature miRNAs and to compare the RNA contents in the Ago1 and Ago2 complexes.

In *An*. *gambiae*, miRNAs were predominantly integrated into Ago1. Ago2 was mainly loaded with siRNAs, but also enclosed a small portion of the miRNAs. Sorting of miRNAs created bias; some miRNAs were primarily associated with Ago1, while others were preferentially integrated into Ago2. The preferential association was essentially unchanged at the six time points after blood feeding. miRNAs were sorted into Ago1 and Ago2 according to their intrinsic structural properties: miRNA duplexes with 5′ end and central mismatches were generally sorted into Ago1, whereas miRNAs with 3′ end mismatches tended to be directed to Ago2. miRNAs associated with Ago1 favored a uridine at the 5′ nucleotide of the guide miRNA. miRNAs in the Ago2 complex were more likely to start with a cytidine at the guide miRNA. These rules for miRNA selective loading are similar to those reported for *Drosophila* ^33^.

When we inspected the miRNAs integrated into Ago1, we found that the Ago association of individual miRNAs was altered at different time points and was not always proportional to their cellular abundance. For example, the amount of miR-989-3p associated with Ago1 was reduced by >8-fold from 24 h PBM to 36 h PBM, when the cellular levels of this miRNA were unchanged. This phenomenon probably reflects additional regulation of miRNA biogenesis and function. During miRNA maturation, the miRNA duplex (~22 bp) is loaded onto an Ago protein to form the RISC. The passenger strand is released, and the guide strand is incorporated into the Ago protein of the mature RISC. However, not all miRNAs are enclosed within Ago proteins. Studies in mammalian cells have shown that Ago proteins are greatly outnumbered by mature miRNAs, and fewer than 10% of the mature miRNAs are bound by Ago proteins^40, 41^. The Ago association of individual miRNAs shows a substantial variation in the same cell line and is not primarily dependent on the miRNAs’ cellular abundance^19^. The degree of Ago association of individual miRNAs varies in different cell types and is altered by the availability of their specific mRNA targets^19^. Overexpression of complementary target mRNAs in human 293 cells leads to enhanced miRNA association with Ago proteins^19^. The altered association of miRNAs with Ago1 that we have observed in *An*. *gambiae* might be influenced by the drastic change in gene expression after a blood meal. The increased expression of mRNA targets of a miRNA may enhance Ago loading or retention of the miRNA in RISC. Reduced expression of the mRNA targets may have the opposite effect.

It is also possible that the Ago association is affected by the availability of miRNAs. Several studies have indicated that Ago proteins compete with other RNA-binding proteins for the binding of some miRNAs^42^. In leukemic blasts, miR-328 controls cell fate by integrating into RISC and also by direct binding to the translational inhibitor hnRNP E2^18^. miR-328 guides RISC to repress translation of the mRNA encoding the survival factor PIM1. In parallel, miR-328 behaves as a RNA decoy and prevents hnRNP2 from binding to the mRNA of CEBPA, a master regulator of myeloid differentiation^18^. TAR DNA-binding protein-43 (TDP-43) is an RNA-binding protein that physically binds miR-1/miR-206 in mouse muscle cells. This interaction is believed to make miR-1/miR-206 unavailable for binding to Ago2 and thus restrains the activity of these two miRNAs^20^.

Although the exact mechanism that regulates the association of miRNAs with Ago is unknown in *An*. *gambiae*, our data clearly indicate that the transcriptome of total cellular small RNA does not accurately reflect the composition of the miRNAs integrated into RISC. A substantial portion of the miRNAs likely exist as RISC-free miRNAs at any time point. Based on our current understanding, integration of miRNA into Ago-containing RISC is essential for miRNA-mediated posttranscriptional regulation^13^. The levels of RISC association are thus more reliably predictive of the inhibitory potential of specific miRNAs than is the miRNA abundance in the total small RNA pool.

Differential association of miRNAs with Ago can be used to identify miRNAs that play robust roles in a biological event. Using this approach, we found that miRNAs originating from the miR-309/miR-2944/miR-286 cluster increased their loading on Ago1 and Ago2 after blood ingestion. This increase was accompanied by correlative changes in the expression of these miRNAs. Depletion of miR-309 profoundly impaired egg production in *An*. *gambiae*. This result echoes a recent study of miR-309 in *Ae*. *aegypti* mosquitoes, which has shown that miR-309 controls ovarian development by inhibiting the expression of the Homeobox gene SIX4^10^.

A more recent study of *Ae*. *aegypti* miRNAs reported a similar increase in the expression of the miR-309/miR-2944/miR-286 cluster in the fat body at 24–48 h after a blood meal^5^. In our experiment, miRNAs were isolated from the abdomens of *An*. *gambiae* mosquitoes. Therefore, the detected changes in miRNA abundance actually reflected the alterations in the overall amounts of miRNAs from all the tissues in the abdomen. Nevertheless, among the 5 miRNAs that were significantly downregulated at 24 h PBM in the fat body of *Ae*. *aegypti*, the RNA levels of miR-184-3p, miR-9c-5p, miR-278-5p, and miR-988-3p in *An*. *gambiae* also decreased at 24 h PBM, suggesting a conserved regulation of miRNA expression after blood ingestion in different mosquito species.

In addition to their roles in blood-feeding and egg development, mosquito miRNAs are also involved in innate immunity. Altered expression of miRNAs have been observed when mosquitoes are infected by malaria parasites or dengue virus^3, 4, 43, 44^. In *Ae*. *aegypti*, miR-375 modulates the immune response against dengue virus by repressing the expression of two immune-related genes, cactus and REL1^45^. Also, miR-281 and miR-252 in *Ae*. *albopictus* have been shown to influence dengue virus replication^46, 47^. A recent study has suggested that miR-305 suppresses the immune response in *An*. *gambiae* as it increases the susceptibility of the mosquito vector to infection by human malaria parasites, and allows for a greater proliferation of the midgut microbiota^48^. In the current study, we identified 13 new miRNAs in *An*. *gambiae* that were expressed at relatively low levels and were significantly enriched by immunoprecipitation of the Ago complexes. The majority of the 13 miRNAs seem to exist exclusively in *Anopheles* mosquitoes, since their orthologs could not be found in mosquitoes of other genera or other organisms. Since human malaria is transmitted by female mosquitoes of the genus *Anopheles*, further investigation of these miRNAs may provide useful insights into the interaction between the malaria parasites and mosquito vectors.

## Methods

### Mosquito rearing and blood feeding

*Anopheles gambiae* (G3 strain) mosquitoes were reared and maintained as previously described^49^. Adult female mosquitoes, 5 days post-emergence, were fed on anesthetized mice. Mosquitoes were collected at 0, 12, 24, 36, 48, and 72 h post-blood meal (PBM). The abdomens of the mosquitoes were dissected on ice, quickly frozen in liquid nitrogen, and stored at −80 °C until use.

### Antibody production

The amino-terminal fragments of *An*. *gambiae* Ago1 (aa 1–120, AGAP011717) and Ago2 (1-aa 1–185, AGAP011537) were individually cloned into the pRSETb vector (Invitrogen) to express His-tagged fusion proteins in *E*. *coli*. The recombinant proteins were purified using a HisPur Cobalt Purification Kit (Thermo Scientific) and then sent to Cocalico Biologicals, Inc. to generate polyclonal antibodies in rabbits. The antisera were affinity-purified using the specific antigens and the AminoLink Plus Immobilization kit (Thermo Fisher Scientific).

### RNA immunoprecipitation and construction of small RNA libraries

For each Ago-IP RNA library, abdomens of 50 adult female mosquitoes were grouped together and homogenized in lysis buffer (50 mM Tris-HCl pH 7.5, 137 mM NaCl, 2 mM EDTA, 1 mM NaF, 0.5% [v/v] NP-40, 0.5 mM dithiothreitol, Halt Protease Inhibitor Cocktail [Thermo Scientific, 1 × ], and RNAsin [Promega, 0.1 U/µl]) and then incubated on ice for 10 min. Supernatants were collected after centrifugation at 16,000 × g, for 15 min at 4 °C. The Ago1 or Ago2 antibodies were first incubated with protein A Dynabeads (Life Technology) for 1 h at 4 °C and then washed three times with W2 buffer (50 mM Tris-HCl pH 7.5, 137 mM NaCl, 1 mM MgCl_2_, 0.1% NP-40). The protein A-coupled antibodies were then incubated with the lysate supernatant for 1 h at 4 °C. The beads were washed three times for 10 min each with the W2 buffer. After immunoprecipitation, RNA on the beads was recovered by protease K digestion (4 mg/ml proteinase K, 100 mM Tris-HCl pH 7.5, 50 mM NaCl, 10 mM EDTA) and phenol-chloroform extraction. Small RNAs of 20–30 nt were size-selected as previously described^29^. The cDNA libraries were constructed using the NEBNext Small RNA Library Prep Set (NEB, E7330S) and run on an Illumina sequencing platform.

For the total small RNA pools, total RNA was isolated from the abdomens of 50 adult female mosquitoes, using TRIzol (Life Technology). RNA size selection and library construction were carried out as for the Ago-IP RNA.

### Real-time PCR

Total cellular RNA and the Ago-associated RNA were isolated as described above. miRNA levels were determined by the miScript PCR System (Qiagen). All the assays were performed in triplicate on an ABI 7300 system (Applied Biosystem). miRNA levels in the total small RNA pools were normalized to the level of 5.8S rRNA in the sample. For the RT-PCR with the Ago-IP samples, internal miRNA controls with invariant expression among libraries were identified by using Normfinder^50^ and geNorm^51^. The combination of miR-100, miR-9c-5p and miR-283 was chosen with a coefficient of variation less than 0.05.

### Basic mapping and annotation

Analysis of small RNA sequencing data was performed using the pipeline of mirPRo^52^ and mirTools v2.0^53^ with adaptation. In brief, the low-quality reads were filtered out using the FASTX-toolkit (http://hannonlab.cshl.edu/fastx_toolkit/index.html), and the 3′ adapter was trimmed from the raw reads with Cutadapt (http://cutadapt.readthedocs.io/en/stable/index.html). The clean reads were first aligned to *An*. *gambiae* pre-miRNA sequences from miRBase. Sequences without a perfect match were then mapped to the *An*. *gambiae* genome (AgamP4, VectorBase) and further annotated using repeats, Rfam, mRNA (AgamP4.0, VectorBase). The raw abundance of the mapped reads was converted to TPM. To filter out sequencing noise, small RNAs with <20 TPM in all libraries were discarded. Heat maps were generated using the R package pheatmap, based on a dendrogram of small RNA expression similarity.

### Prediction of novel miRNAs

Novel miRNAs were predicted using miRdeepv2^54^ and the MIREAP algorithm (http://sourceforge.net/projects/mireap). All the unannotated reads with more than 20 reads in at least one of the Ago-IP libraries were taken as input for this analysis. Novel miRNAs were retained if predicted by both algorithms and the free energy of their putative hairpin structure was less than −20 kcal/mol.

### Conservation analysis of miRNAs

To explore the conservation of novel miRNAs, their orthologs were searched against the genomes of *D*. *melanogaster*, *Ae*. *aegypti*, *Ae*. *albopictus*, *C*. *quinquefasciatus*, and 19 other *Anopheles* species. The miRNA precursors were mapped to each genome by reciprocal BLAST^55^. The miRNA is considered to have an ortholog if blasting with its precursor sequence returns the top hit with an identity match of ≥80% and e-value ≤ 0.1, and vice versa.

### Differential expression of miRNAs

To identify the differentially expressed miRNAs, sequence reads with 3′-end variation were also included in the counts. Comparisons were made between each time point after blood feeding and the non-blood-fed control. The DEGSeq package (version 1.10.0) was used to assess miRNA differential expression, utilizing a MARS (MA-plot-based method with Random Sampling model) method^56^. For each gene, the p-value, q-value and fold changes were calculated. The p-value was calculated with two-sided z-test comparing expression between two conditions. This method is widely used in RNA-seq studies lacking biological replicates^57, 58^.

### Analysis of miRNA sorting into distinct Ago complexes

To study the sorting of miRNAs between Ago1 and Ago2, we manually curated the mature miRNA star and non-star strands based on the sequencing data from the total RNA pools as previously described^59^. For miRNA precursors that had two detectable mature miRNAs, we calculated the mean expression value of all time points for the two strands; the more abundant one was annotated as the non-star strand, whereas the less abundant one was annotated as miRNA*. For those precursors with only one detectable mature miRNA, this miRNA was treated as the non-star sequence.

To explore the sorting rules for miRNAs, the read counts of each miRNA were converted into the percentage of miRNAs in the libraries. The enrichment of miRNA* was determined by Fisher’s exact test, comparing the Ago-associated miRNA* with that from the total RNA, as previously described^60^. The base pairing probability of each nucleotide was calculated based on the secondary hairpin structures from RNAfold^61^. For each Ago-enriched mature miRNA, we used its pre-miRNA sequence as input for RNAfold. The most abundant isoform was selected as the mature miRNA sequence and might be different from the miRBase annotation.

### miRNA sequence variation

Analyses of the miRNA sequence variations were conducted using mirPRo^52^. In brief, with the most abundant sequence as reference, the miRNA sequence variations were classified as template-directed shift and non-template modifications at the 5′ end and 3′ end. The template-directed variations were defined as 1, 2, and 3-nt shifts. The non-template variations included single-nucleotide addition, dual-nucleotide addition and so on. All the data were collected from the output of mirPRo for further analysis.

### Functional study of miRNAs in mosquitoes

Antagomirs of miR-309 were purchased from GENEPHARMA (Shanghai, China) and each designed as the reverse complement of the mature miR-309 sequence. Mosquitoes were anesthetized on ice at 12 h after eclosion, and antagomirs were microinjected into the thorax at a dose of 15 pmol. Mosquitoes were then maintained on 10% sugar for 3 days before further analysis.

## Electronic supplementary material

Supplementary Information

Table S1

Table S2

Table S3

Table S4
