# Supplementary material for: Association of microRNAs with Argonaute proteins in the malaria mosquito Anopheles gambiae after blood ingestion
Source: Sci Rep. 2017 Jul 26;7:6493. doi: 10.1038/s41598-017-07013-1 (PMC5529372; doi:10.1038/s41598-017-07013-1)
Supplement: Supplementary file 1 — Supplementary Information [file 41598_2017_7013_MOESM1_ESM.pdf]

Association of microRNAs with Argonaute proteins in the malaria mosquito *Anopheles gambiae* after blood ingestion

Xiaonan Fu[1], George Dimopoulos[2], Jinsong Zhu[3,*]

[1]The Interdisciplinary PhD Program in Genetics, Bioinformatics, and Computational Biology, Virginia Tech, Blacksburg, Virginia, USA

[2]W. Harry Feinstone Department of Molecular Microbiology and Immunology, Bloomberg School of Public Health, Johns Hopkins University, Baltimore, Maryland, USA.

[3]Department of Biochemistry, Virginia Tech, 340 West Campus Drive, Blacksburg, Virginia 24061, USA

[*] Correspondence and requests for materials should be addressed to J.Z. (email: zhujin@vt.edu)

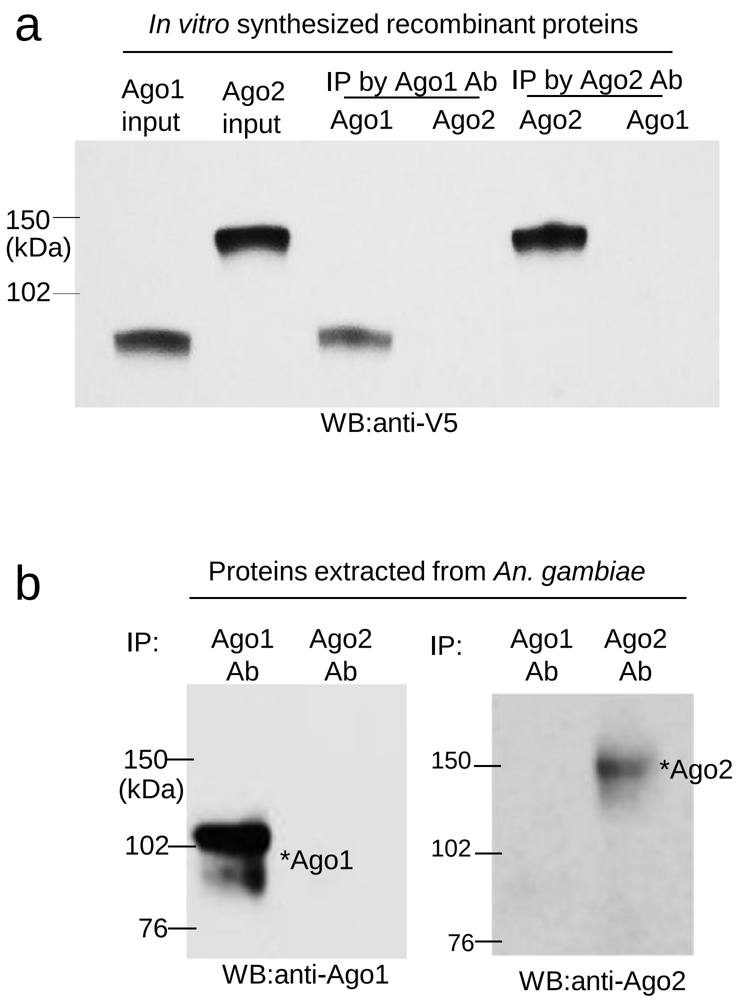

Supplementary Figure S1. Specificity of the Argonaute antibodies. (**a**) Immunoprecipitation of *in vitro* synthesized Ago proteins. The Ago1-V5 and Ago2-V5 fusion proteins were expressed using the TNT translation system. The C-terminal end of Ago1 was not included in the fusion. After incubation with the Ago1 or Ago2 antibodies, the pellets were subjected to Western Blot analysis using an antibody for the V5 tag. IP: immunoprecipitation. WB: Western Blot. (**b**) Immunoprecipitation of Ago proteins extracted from mosquito abdomens (5 days after eclosion). Protein extracts were incubated with the Ago antibodies. Ago proteins in the pellets were detected in Western Blot analysis by the Ago antibodies.

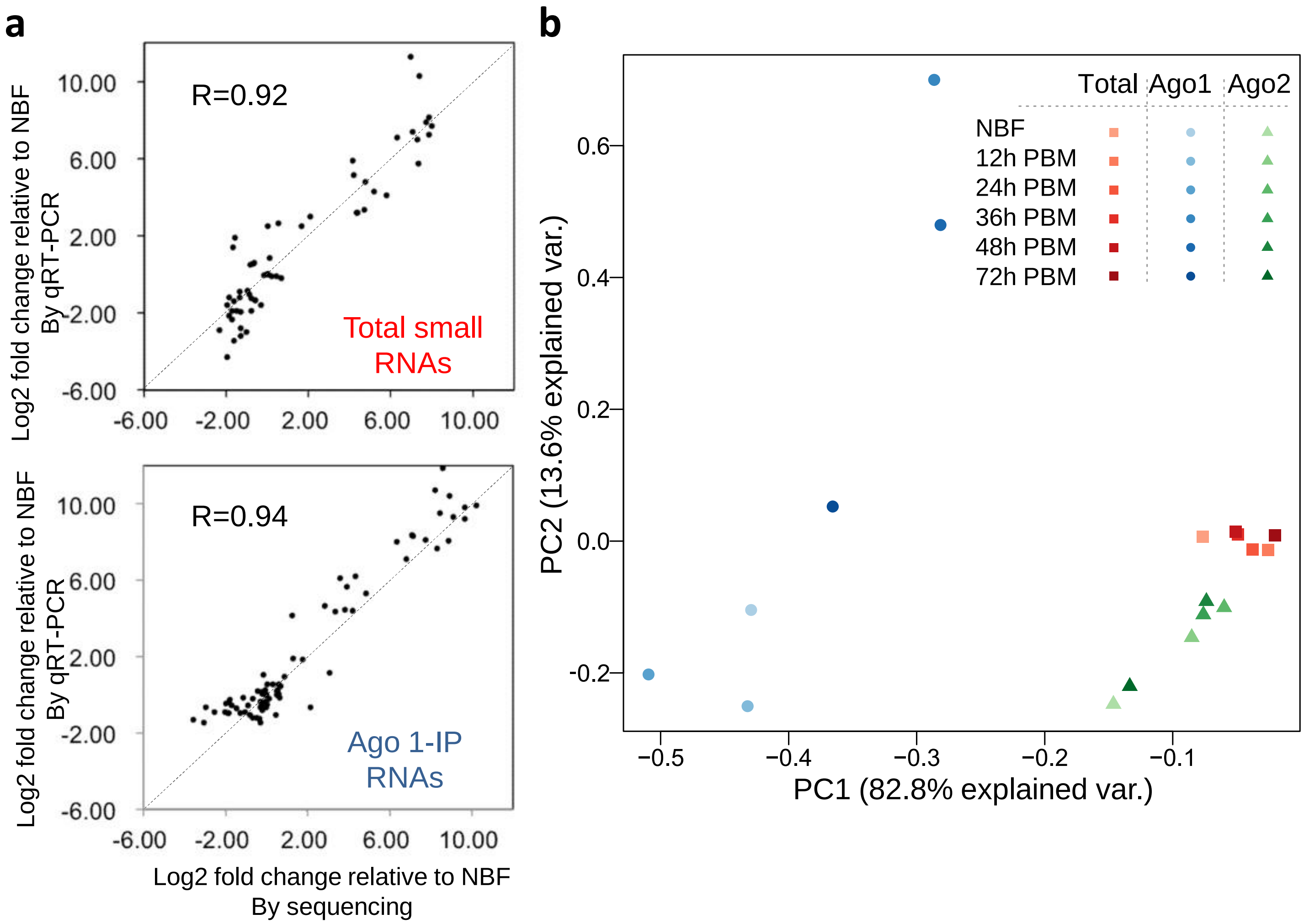

Supplementary Figure S2. Validation of the RNA-seq results. (**a**) Correlation of expression fold changes determined using qPCR and small RNA sequencing. Ten miRNAs with considerable changes in RNA abundance were randomly picked for this analysis. Their expression levels after blood feeding were compared with those of the non-blood-fed controls (NBF). (**b**) Principal component analysis (PCA) shows PC1 (82.8% explained) versus PC2 (13.6% explained) of all the expressed miRNAs above the detected level (TPM ≥20, at more than one time point).

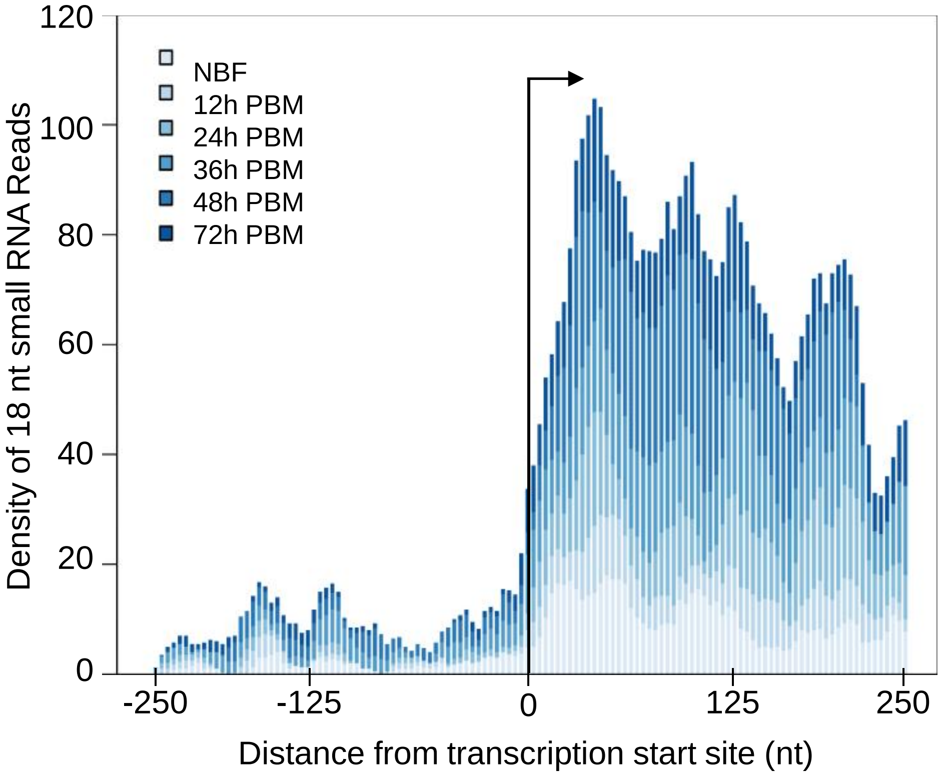

Supplementary Figure S3. Genome-wide distribution of small RNA reads of 18 nt in length with respect to transcription start sites. RNA reads of 18 nt in length from the total small RNA pools were mapped to the *Anopheles gambiae* genome (AgamP4, VectorBase) without mismatches. The resulting set was further filtered to remove the annotated non-coding RNAs. The remaining reads counts were plotted against the distance to the transcription start sites of annotated genes (AgamP4.5, VectorBase). The black vertical line indicates the transcription start site, and the black arrow depicts the direction of transcription. The stacked bars represent windows of 4 nt.

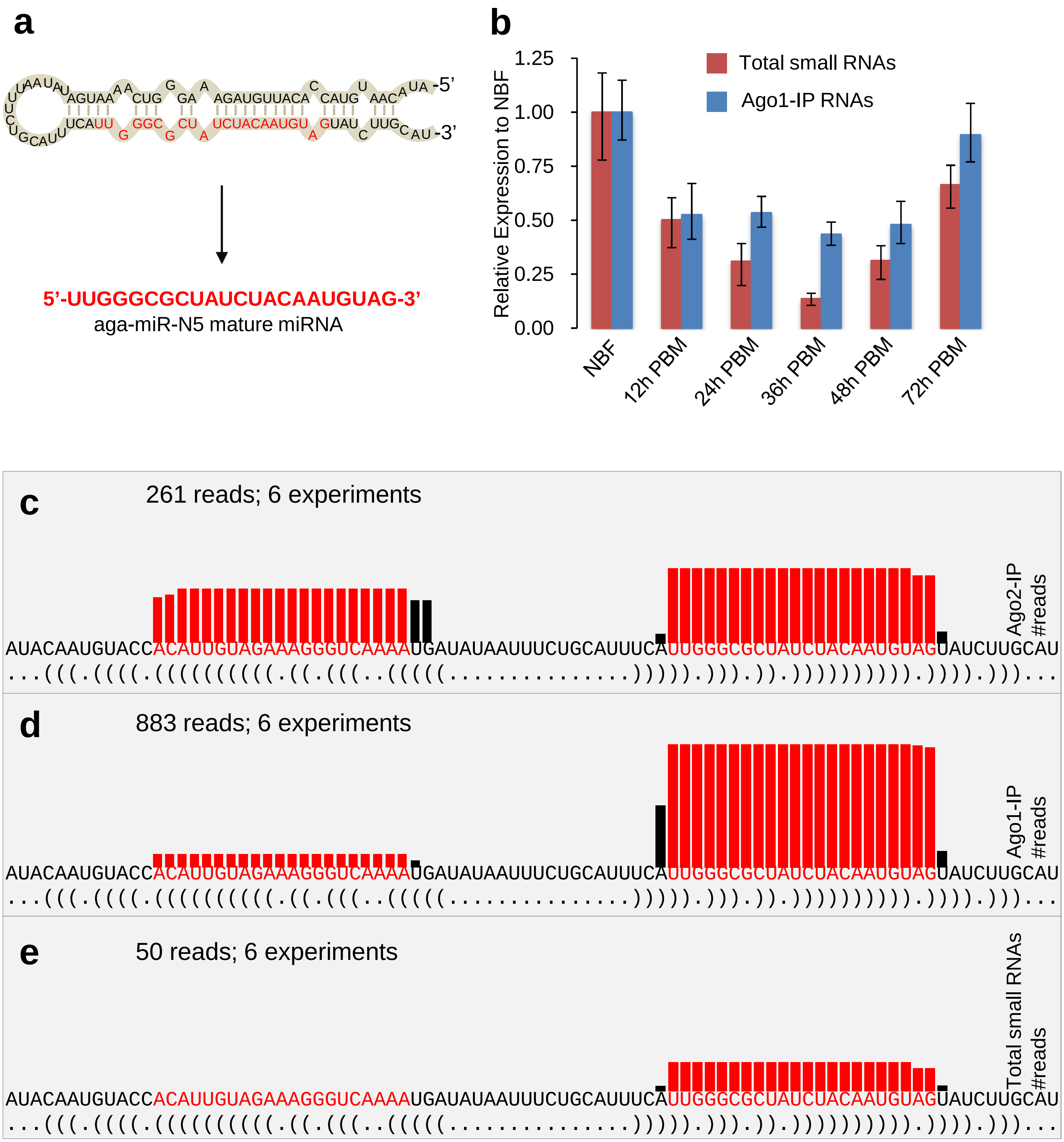

Supplementary Figure S4. Detection of aga-miR-N5 in *An. gambiae*. (**a**) The RNA secondary structure of aga-mir-N5 predicted by RNAfold. Mature sequence of miR-N5 is highlighted in red. (**b**) qRT-PCR verification of miR-N5 in the Total and Ago1-IP RNA pools. Error bar represents standard deviation. (**c-e**) Distribution of the numbers of sequence reads detected in the Ago2-IP, Ago1-IP and Total small RNA libraries. Red bars represent the most-abundant mature miRNA. Black bars represent the variants of mature miR-N5. The pre-miRNA sequence and its secondary structure in dot-bracket notation (from RNAfold) are included at the bottom of the image.

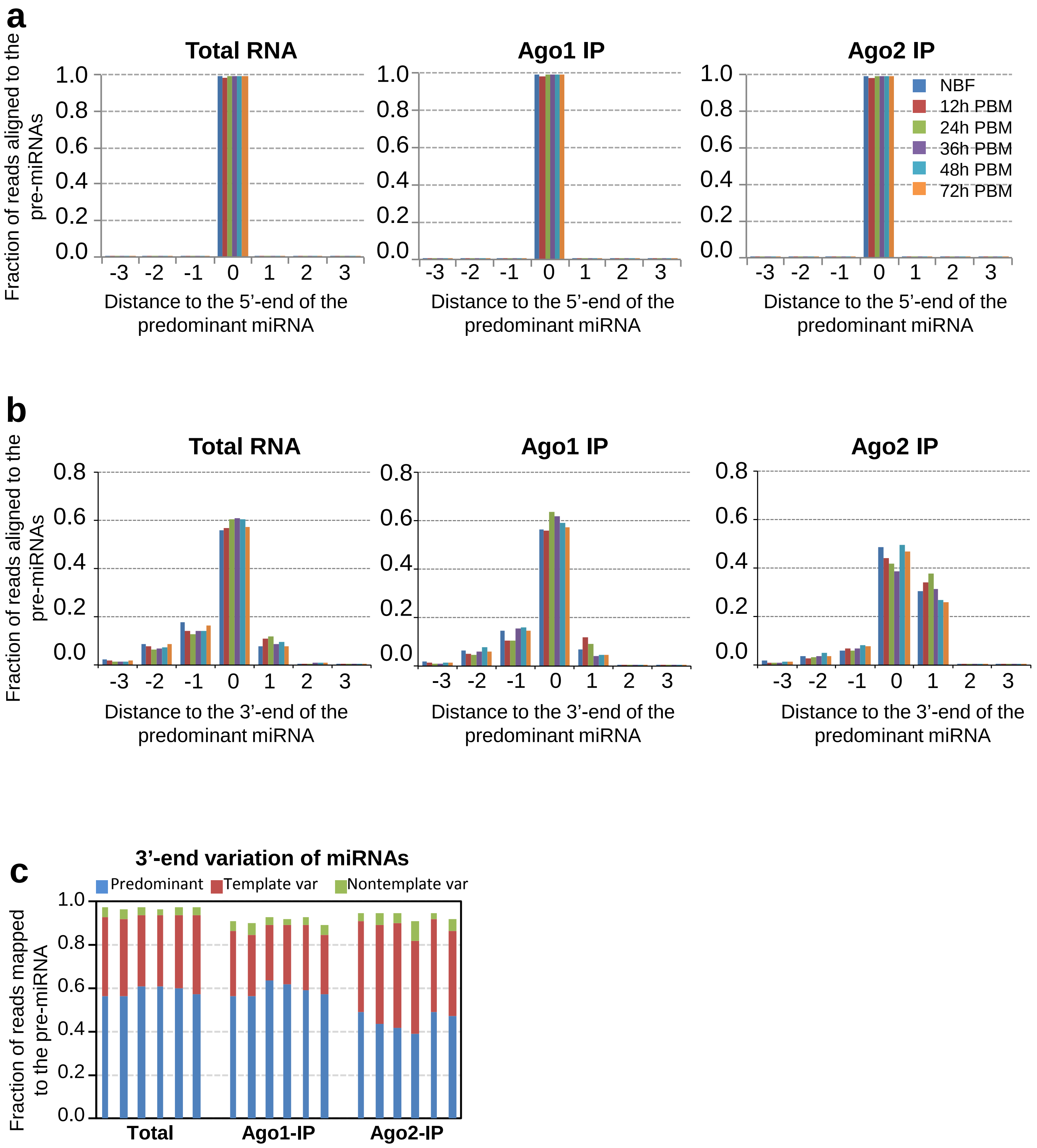

Supplementary Figure S5. Sequence heterogeneity at the ends of miRNA. 5'-end (**a**) or 3'-end (**b** and **c**) sequences were analyzed to identify sequence variations in miRNAs derived from the Total, Ago1-IP and Ago2-IP libraries. Template var, template-directed variation; nontemplate var, nontemplate-directed variation.

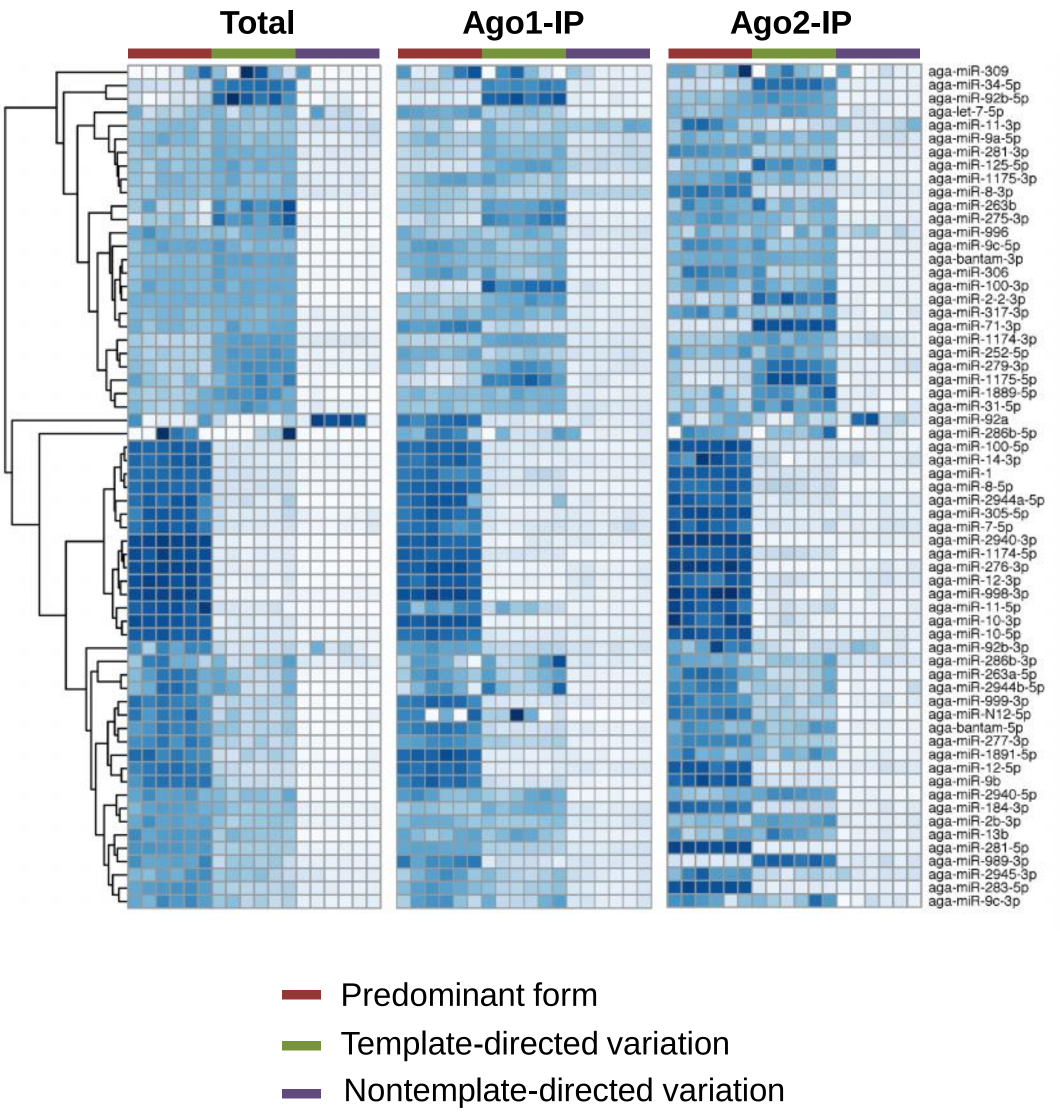

Supplementary Figure S6. Sequence variations of miRNA at 3'-end. Heat maps compare the 3'-end miRNA sequence variations among RNA reads derived from different libraries. Each small square represents an RNA library from samples collected at one of the 6 time points after blood feeding. The predominant mature miRNAs, template-directed and nontemplate-directed variations were identified in each library.

a **aga-miR-989-3p**

5'-UGUGAUGUGACGUAGUGGUACU 3' non-template directed variation
5'-UGUGAUGUGACGUAGUGGUAC**C** 3' template-directed variation
5'-UGUGAUGUGACGUAGUGGUAC Predominant form

5'-GGACAGUACAGUGGCCACGGGGGUACGCCGCUACGUUG
CUUUCACUUGGUACGCUCGUAUAGUAUAGAGCUAAAAGUA
AACAUAGAUCCA**UGUGAUGUGACGUAGUGGUAC**CCUCCCG
UGUCUAUCACAAC pre-miR-989

b

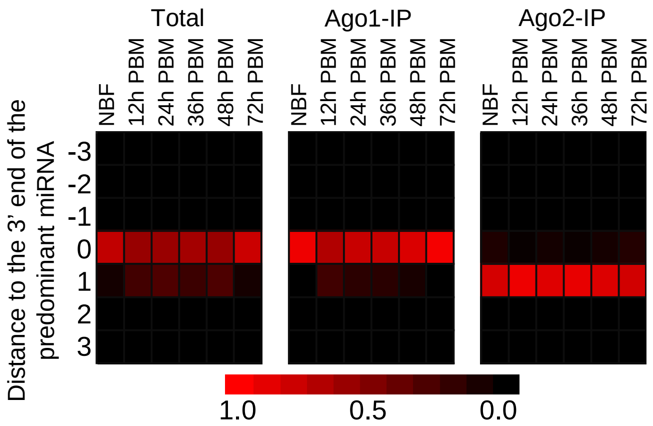

Supplementary Figure S7. Length divergence at the 3'-end of aga-miR-989-3p. (**a**) Sequences of iso-miRs. (**b**) Preferential association of the 23-nt miR-989-3p variant with Ago2. Sequence reads were grouped by their 3'-end and compared with the length of the predominant mature aga-miR-989-3p (0). The percentage of 3'-end variations in each RNA library is in black/red scale.

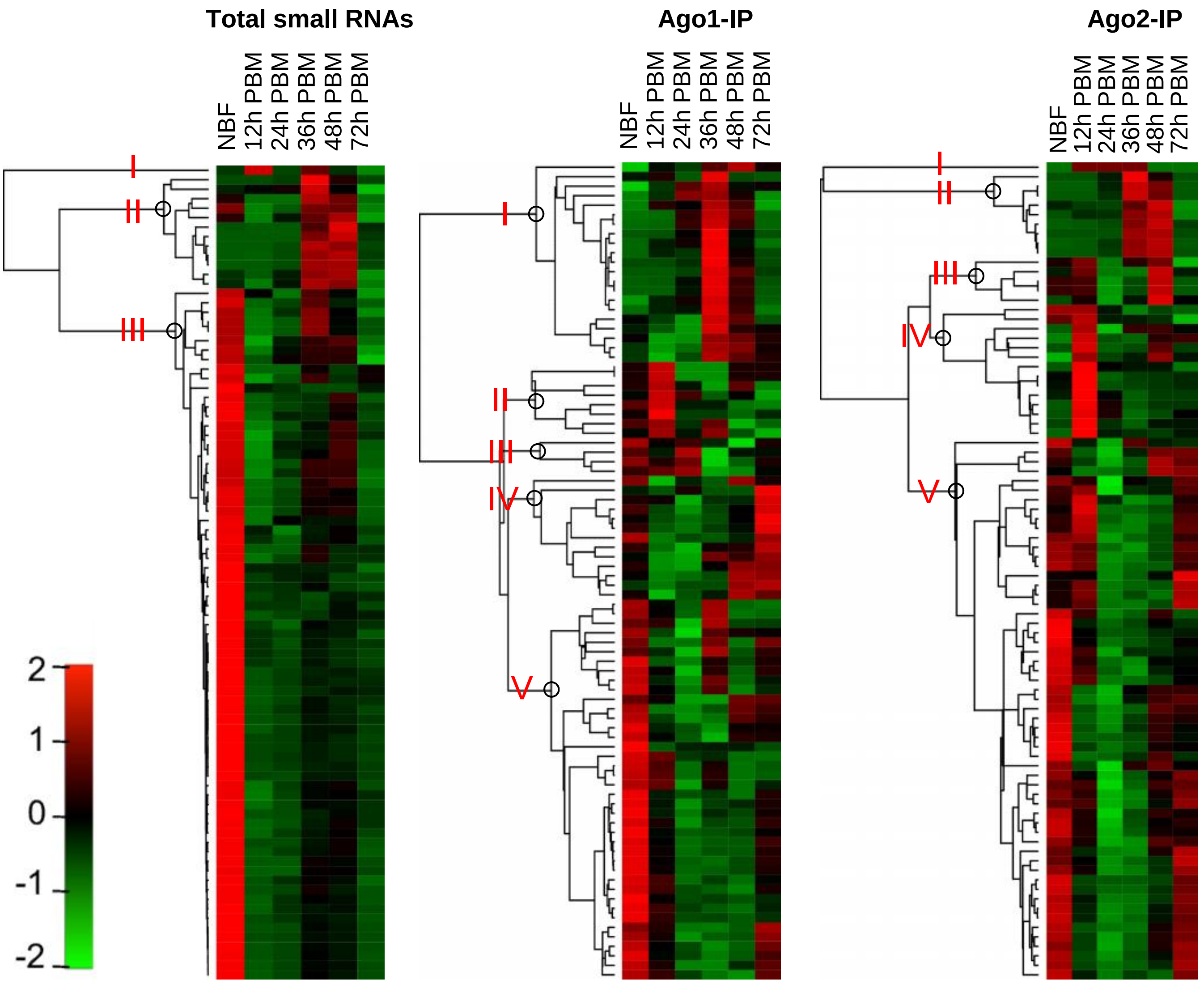

Supplementary Figure S8. Expression profiles of *An. gambiae* miRNAs after blood feeding. Hierarchical clustering of total miRNAs and the Ago-associated miRNAs over the time course. The normalized reads of miRNAs were Z-score transformed prior to analysis. miRNAs were clustered using correlation distance with complete linkage. The level of miRNA expression is depicted using a green-red color key.

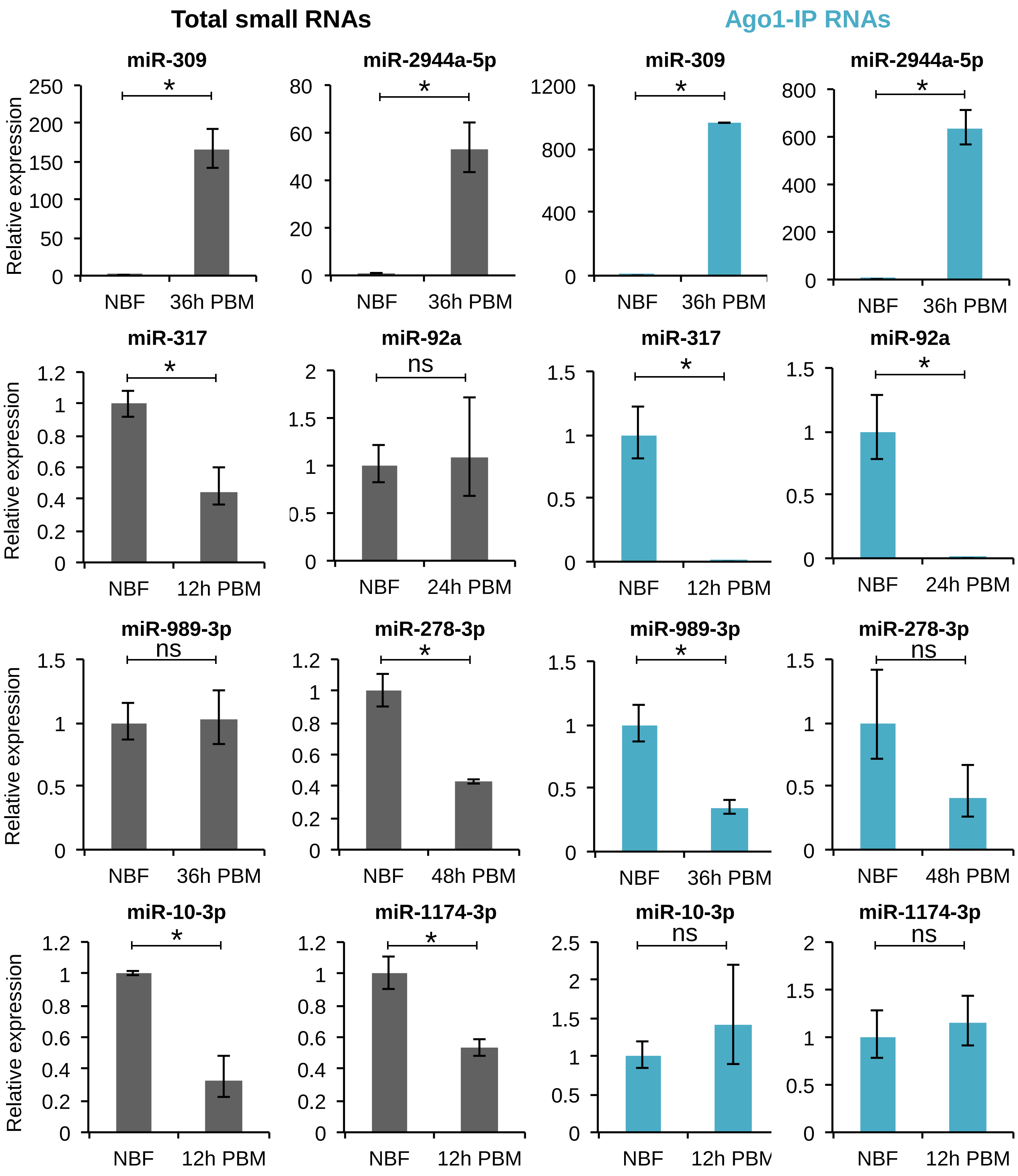

Supplementary Figure S9. qRT-PCR validation of the abundance and Ago1 loading of selected miRNAs. The levels of miRNA in the total small RNA pools and the Ago1-IP pools were measured using real-time PCR. Results were the mean ± standard deviation of three independent experiments. Statistical significance was calculated using unpaired t tests. (*, *p* value < 0.05; ns, *p* value ≥ 0.05).

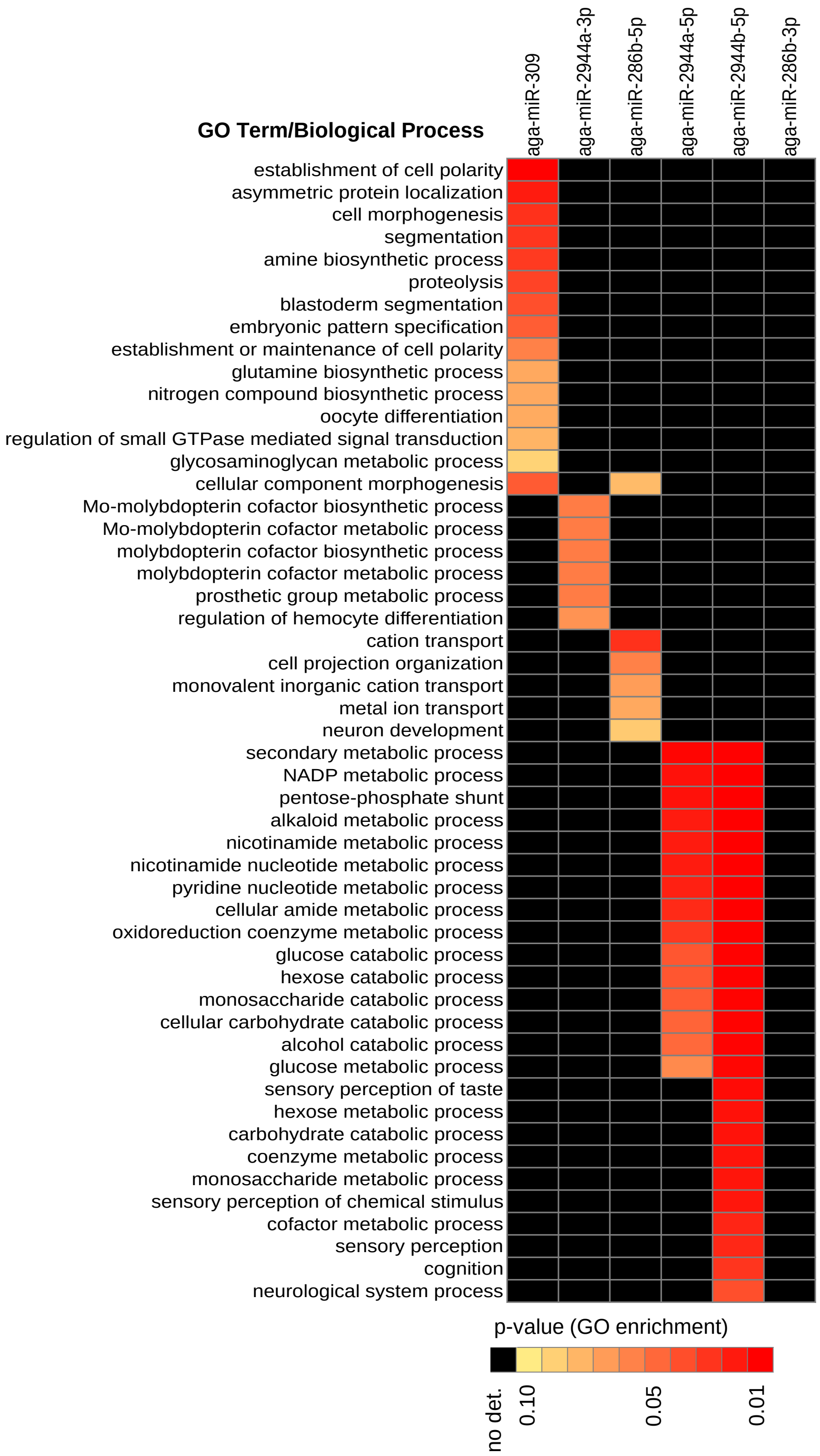

Supplementary Figure S10. Functional enrichment analysis of the predicted targets of miR-309/2944/286. mRNA targets were predicted by bioinformatics analysis. Heat map illustrates the overrepresented "biological process" gene ontology terms associated with the putative target genes of miR-309/2944/286.
